# Association between irregular daily routine and risk of incident stroke and coronary heart disease in a large Japanese population

**DOI:** 10.1038/s41598-022-20019-8

**Published:** 2022-09-21

**Authors:** Takahiro Yoshizaki, Junko Ishihara, Ayaka Kotemori, Yoshihiro Kokubo, Isao Saito, Hiroshi Yatsuya, Kazumasa Yamagishi, Norie Sawada, Motoki Iwasaki, Hiroyasu Iso, Shoichiro Tsugane, The JPHC Study Group

**Affiliations:** 1 Department of Food and Life Sciences, Faculty of Food and Nutritional Sciences, Toyo University, 1-1-1 Izumino, Itakura-Machi, Oura-Gun, Gunma, 374-0193 Japan; 2 Department of Food and Life Science, School of Life and Environmental Science, Azabu University, 1-17-71 Fuchinobe, Chuo-Ku, Sagamihara City, Kanagawa 252-5201 Japan; 3 Epidemiology and Prevention Group, Center for Public Health Sciences, National Cancer Center, 5-1-1 Tsukiji, Chuo-Ku, Tokyo, 104-0045 Japan; 4 Department of Preventive Cardiology, National Cerebral and Cardiovascular Center, 6-1 Kishibe-Shimmachi, Suita, Kishibe-Shimmachi, Suita City, Osaka 564-8565 Japan; 5 Department of Public Health and Epidemiology, Faculty of Medicine, Oita University, 1-1 Idaigaoka, Hasama-Machi, Yufu City, Oita 879-5593 Japan; 6 Department of Public Health and Health Systems, Nagoya University Graduate School of Medicine, 65 Tsurumai-Cho, Showa-Ku, Nagoya City, Aichi 466-8550 Japan; 7 Department of Public Health Medicine, Faculty of Medicine, and Health Services Research and Development Center, University of Tsukuba, 1-1-1 Tennodai, Tsukuba City, Ibaraki 305-8575 Japan; 8 Public Health, Department of Social Medicine, Osaka University Graduate School of Medicine, 2-2 Yamadaoka, Suita City, Osaka, 565-0871 Japan

**Keywords:** Nutrition, Public health, Epidemiology

## Abstract

Circadian misalignments have been linked to adverse cardiometabolic outcomes. However, the association between irregular daily routine and the risk of cardiovascular disease (CVD) remains unknown. We examined this association in a prospective study in Japan. The study included 78,115 Japanese participants aged 45–74 years. The self-reported daily routine was evaluated using the question, ‘Is your daily routine or activity schedule regular?’ The response (yes/no) was obtained as a binary variable. Cox proportional hazard regression analysis was used to estimate the hazard ratios and 95% confidence intervals for the association between an irregular daily routine and CVD incidence risk. Among the participants, 23.7% reported an irregular daily routine. During the mean follow-up period of 13.3 years, we observed 4641 CVD events. An irregular daily routine was significantly associated with increased risks of CVD and total stroke in women, but not in men. This positive association between an irregular daily routine and the risk of CVD was weak in the high vegetable and fruit consuming population. An irregular daily routine is positively associated with the risk of incident CVD, especially in women. These associations may be weak in populations that consume a diet rich in vegetables and fruits.

## Introduction

Current lifestyles in developed countries have been affected and modified by multiple aspects of the 24/7 (24 h a day and 7 days a week) society. In society, daily exposure to artificial light at night, multimedia device usage in bedrooms, staying up late, skipping breakfast, late dinner time, gaps between non-working and working day schedules, working overtime, and flexible work arrangements are prevalent. In such an environment, circadian misalignments (i.e. inappropriate behavioural cycles relative to biological rhythms) may be a general phenomenon. They could occur by exposure to an irregular daily routine, mainly determined by daily sleep and meal schedules^1^.

Recently, several experimental studies have shed light on the pivotal role of circadian alignment between the circadian clock in the suprachiasmatic nucleus and the sleep–wake cycle, light–dark cycle, and other peripheral clocks as a potential benefit against health problems^2–9^. Moreover, because of the circadian patterns of all cardiovascular functions and time-of-day variations in adverse cardiovascular events, a large degree of circadian misalignment (e.g. day-night reversal) has been reported to be associated with cardiovascular disease (CVD) risk in a meta-analysis of cohort studies^10–13^.

An irregular daily routine could be associated with CVD risk. For example, in the Multi-Ethnic Study of Atherosclerosis, regular sleep–wake schedules were assessed based on seven-day actigraph data collected under a free-living situation and were associated with increased levels of cardiometabolic outcomes, such as obesity, hypertension, and metabolic syndrome, as well as with increased risk of CVD in a cross-sectional^14,15^ and prospective study (4.9-year follow-up period) performed in a large population of older adults^16^. Moreover, breakfast frequency has also been associated with the risk of CVD^17–20^. Those who consider themselves as following an irregular daily routine might be more likely to indulge in behaviours, such as the instability of sleep–wake and eating schedules^21^, that lead to circadian misalignments. However, to the best of our knowledge, the association between a self-reported irregular daily routine, which is mainly indicative of the stability of sleep–wake and meal schedules, and the CVD risk is still unknown.

Therefore, this study aimed to elucidate the association between a self-reported irregular daily routine and the risk of incident CVD in a large-scale, middle- and older-aged population-based prospective study. Furthermore, some individuals with an irregular daily routine cannot change their behavioural patterns or schedules, even if they want to, because of work-related, physical, or environmental reasons. Because dietary habits are one of the changeable factors and vegetable and fruit consumption has been associated with CVD risk^22^, this may lead to new insights into these populations. Therefore, we also investigated whether other potential factors, such as vegetable and fruit consumption or demographic characteristics, could modify the association between a self-reported irregular daily routine and CVD risk. We hypothesised that an irregular daily routine was associated with the increased risk of CVD and that these associations were modified by vegetable and dietary intake or demographic characteristics.

## Materials and methods

### Study population

The Japan Public Health Center-Based Prospective Study (JPHC Study) is a prospective study performed using a population-based sample of 140,420 Japanese adults (68,722 men and 71,698 women) in two cohorts based on public health centre (PHC) areas: Cohort I (started in 1990, five PHC areas) and Cohort II (started in 1993, six PHC areas).

The residents of these 11 PHC areas were identified through population registries managed by local municipalities. A previous study has presented the details of the JPHC Study protocol^23^. Since participants from the Tokyo and Osaka areas were excluded from the present study due to unavailable incidence data, 116,896 participants (57,713 men and 59,183 women) from nine PHC areas were considered eligible for follow-up. Informed consent was obtained from each participant implicitly when they completed the baseline questionnaire, where the study purpose and follow-up procedures were described. This study followed the tenets of the Declaration of Helsinki and was approved by the institutional review boards of Azabu University, Toyo University, and the National Cancer Center, Tokyo, Japan.

A self-administered questionnaire was distributed to all residents in the study areas for Cohorts I and II. The questionnaire included items that captured demographic characteristics, medical history, smoking and drinking habits, and a simplified version of dietary habits. A 5-year follow-up survey was conducted for Cohort I in 1995 and Cohort II in 1998 using a questionnaire that included items regarding irregular routine in daily life and demographic characteristics, in addition to a more comprehensive food frequency questionnaire (FFQ). Therefore, we used these questionnaires as the starting point to assess the association between irregular daily routine and CVD incidence risk.

**Figure 1:**
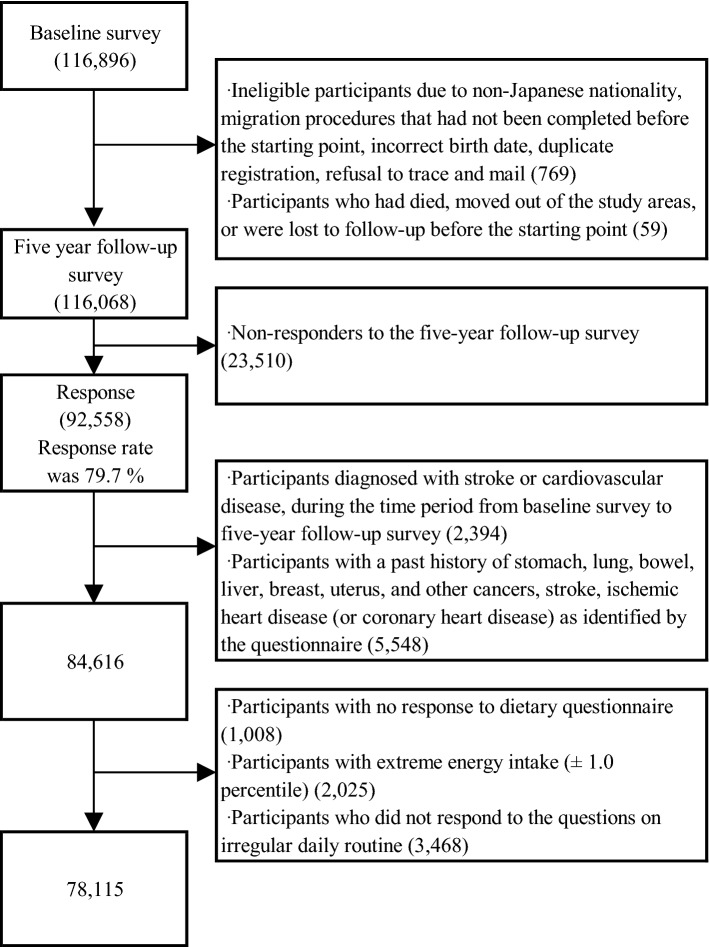
Flowchart of study participants.

We excluded participants with non-Japanese nationality, migration procedures that had not been completed before the starting point, incorrect birth date, duplicate registration, and those who refused follow-up, died, moved out of the study areas, or were lost to follow-up before the starting point. Finally, 116,068 participants were eligible for this study. Among them, 92,558 participants responded to the 5-year follow-up questionnaire, yielding a response rate of 79.7%, and were included. Among these participants, we excluded those who were diagnosed with CVD in the period from the baseline survey to the 5-year follow-up survey (n = 2394) and those who had a past history of cancer (e.g. stomach, lung, bowel, liver, breast, uterine cancer, or other cancers), stroke, and ischaemic heart disease (or coronary heart disease [CHD]), as identified by the questionnaire (n = 5548). Furthermore, 1008 participants who did not complete the diet section of the questionnaire, 2025 participants with extreme total energy intake (lower and upper 1.0 percentile for men and women: 785 and 5096 kcal/day, and 656 and 4472 kcal/day, respectively), and 3468 participants who did not respond to the questions on irregular daily routine were excluded. Finally, 78,115 participants (36,210 men and 41,905 women) were included in the statistical analyses (Fig. 1).

### Assessment of irregular daily routine and dietary intake

The self-reported irregular daily routine was assessed by the response to the question, ‘Is your daily routine or activity schedule regular?’ in the questionnaire. The possible response to this question was ‘yes’ or ‘no’. Participants who responded with ‘no’ were considered to have an irregular daily routine; participants who responded with ‘yes’ were included as the reference group. Dietary intake was assessed using the FFQ, consisting of 138 food and beverage items. The 5-year follow-up questionnaire was based on nine frequency categories (‘almost never’ to ‘seven or more times per day’) for vegetables and fruits and nine frequency choices (‘almost never’ to ‘10 or more glasses per day’) for juices. Standard portion sizes for each food item were categorised as follows: small (50% smaller than standard), medium (same as standard), and large (50% larger than standard). The quantity (g/day) of consumed vegetables and fruits was calculated from the data obtained. The FFQ for assessing vegetable and fruit consumption has already been validated in previous studies^24,25^.

### Potential confounding factors

Potential confounding factors included age, sex, body mass index (BMI) (quartile), smoking status (never smoker, ex-smoker, or current smoker), sleep duration (6–8 h/day, ≤ 5 h/day, ≥ 9 h/day), breakfast (eating every day or other frequencies), working hours, perceived mental stress (low, medium, or high), living arrangement (living alone or others), job type (agriculture, forestry, fishery, or self-employed; salaried or professional; housework or unemployed), study area, alcohol intake (0, 1–150, 150–300, or ≥ 301 g/week), quartiles of metabolic equivalent task-hours per day (METs), total energy intake, vegetable, fruit, fish, meat, and sodium intake. The consumption of each food group was adjusted by the total energy intake using the residual method^26^.

### Follow-up survey

Participants in Cohorts I and II were followed from 1995 to December 31, 2009, and from 1998 to December 31, 2012, respectively. Information about any changes in residence status was obtained annually from data on moving out of the area, identified by residency registration inside and outside the PHC areas. All mortality data of participants in the residential registry were forwarded to the Ministry of Health, Labour and Welfare and were coded for inclusion in the National Vital Statistics. Since residency and death registrations are required by the Basic Resident Registration Law and Family Registration Law, respectively, data from each registration were considered complete.

### Ascertainment of stroke and CHD incidence

The medical records were reviewed by hospitals or PHC physicians in each registered major hospital within the PHC areas^27,28^. Diagnoses of stroke were based on focal neurologic symptoms and confirmed using computed tomography scan and/or magnetic resonance imaging according to the criteria of the National Survey of Stroke^29^. Diagnoses of myocardial infarction were confirmed in the medical records according to the criteria of the MONICA project^30^. Sudden cardiac death was defined as death of unknown origin that occurred within 1 h of the onset of the event. These medical data were extracted according to cohort-specific registration forms.

For the analyses, only the first-ever CVD events during the follow-up were regarded as incident CVD. If one person suffered both stroke and CHD, CVD was defined, whichever occurred first. Person-years of follow-up were calculated for each participant from baseline to the date of death, diagnosis, emigration from the PHC area, or end of the follow-up period (December 31, 2009, or December 31, 2012), whichever occurred first. Participants lost to follow-up were censored at the last confirmed date of their presence in the PHC area. A total of 1,038,087 person-years for the CVD analysis was accrued.

### Statistical analysis

All statistical analyses were divided by sex, and the participants were divided into two groups according to their daily routine: the regular and irregular groups. Hazard ratios (HRs) and 95% confidence intervals (CIs) were calculated using the Cox proportional hazards model, according to the SAS PHREG Procedure (SAS Institute, Inc., Cary, NC, USA). Multivariable Cox regression models with a covariate adjustment approach were used: model l included age at baseline and study area; model 2 included model 1 plus covariates, including living arrangement (living alone, other, or missing), alcohol intake (0, 1–150, 151–300, 301–450, and ≥ 451 g/week), cigarette smoking status (current [< 20 or ≥ 20 cigarettes/day], never, former, or missing), perceived mental stress (low, medium, high, or missing), working hours (< 5 h, ≥ 5 h and < 9 h, ≥ 9 h, or missing), job type (agriculture, forestry, fishery, and self-employed; office work, profession, and other work; housework and unemployed; or missing), breakfast (eating every day, other frequencies, or missing), sleep duration (≤ 5 h, ≥ 6 h and ≤ 8 h, ≥ 9 h, or missing), quartile of BMI, METs, and dietary factors (i.e. quartiles of energy intake and energy-adjusted dietary intakes of vegetables, fruits, meat, fish, and sodium). Missing covariate data were adjusted using dummy variables. Since hypertension, hypercholesterolaemia, and diabetes mellitus could be considered potential mediators of the association between an irregular daily routine and the increased risk of CVD, we included multivariable analysis data concerning the medication use for hypertension, hypercholesterolaemia, and diabetes mellitus in Table S1.

Since a significant inverse association between vegetable and fruit consumption and CVD risk has been reported^22^, we further explored the interaction between an irregular daily routine and vegetable and fruit consumption (dichotomised variable) on CVD risk by adding cross-product terms into the multivariable model. Moreover, stratified analyses according to selected demographic characteristics (i.e. BMI, living arrangement, smoking and drinking habits, working hours, type of job, total physical activity, perceived mental stress, sleep duration, and skipping breakfast) were also conducted to determine the heterogeneity of association between an irregular daily routine and CVD risk. A two-tailed *p*-value of < 0.05 was considered statistically significant.

### Ethics approval and consent to participate

Informed consent was obtained from each participant implicitly when they completed the baseline questionnaire, where the study purpose and follow-up procedures were described. The study protocol was approved by the institutional review boards of Azabu University, Toyo University, and the National Cancer Center, Tokyo, Japan.

## Results

The associations between a self-reported irregular daily routine and demographic characteristics are shown in Table 1. The self-reported irregular daily routine was significantly associated with sex, and the percentage of individuals with an irregular daily routine was 26.0% and 21.7% among men and women, respectively. In both men and women, an irregular daily routine was significantly associated with lower age, lower consumption of vegetables, fruits, fish, and fibre, higher BMI, and higher consumption of meat. Both men and women with an irregular daily routine were more likely to live alone, smoke and drink, work long hours, have higher levels of mental stress, sleep ≤ 5 h a day, and skip breakfast. The percentage of the job type category ‘office work, profession, or other work’ and ‘homemaker or unemployed’ was the highest in men and women, respectively, with an irregular daily routine.

**Table 1:** Baseline characteristics according to self-reported irregular daily routine.

	Men		p values*	Women		p values*
	Regular	Irregular		Regular	Irregular	
Number of participants	26,801	9409		32,822	9083	
Age (years)	57 ± 8	55 ± 7	< 0.001	57 ± 8	56 ± 7	< 0.001
BMI (kg/m^2^)	23.5 ± 2.9	23.8 ± 3.2	< 0.001	23.4 ± 3.1	24.0 ± 3.5	< 0.001
Living alone^†^ (%)	696 (2.6)	480 (5.2)	< 0.001	1944 (6.0)	765 (8.6)	< 0.001
Non-smoker^†^ (%)	9568 (37.9)	2723 (30.9)	< 0.001	29,245 (95.3)	7616 (90.9)	< 0.001
Non-drinker (%)	7199 (26.9)	2407 (25.6)	0.016	27,518 (83.8)	7299 (80.4)	< 0.001
**Job type**^**†**^						
Farming, forestry, fishery, self-employed (%)	4986 (23.4)	2448 (31.0)	< 0.001	2555 (9.3)	1302 (17.0)	< 0.001
Office work, profession, other work (%)	13,616 (64.0)	4677 (59.2)		9082 (33.0)	2547 (33.2)	
Homemaker, unemployed (%)	2672 (12.6)	777 (9.8)		15,892 (57.7)	3824 (49.8)	
**Working hours**^**†**^						
< 5 h (%)	3574 (14.3)	1027 (11.8)	< 0.001	9867 (33.2)	2209 (27.1)	< 0.001
≥ 5 h and < 9 h (%)	17,402 (69.5)	4831 (55.3)		16,865 (56.7)	4386 (53.8)	
≥ 9 h (%)	4075 (16.3)	2871 (32.9)		2988 (10.1)	1558 (19.1)	
**Perceived mental stress**^**†**^						
Low or middle (%)	22,925 (86.0)	6502 (69.7)	< 0.001	28,230 (86.6)	6253 (69.4)	< 0.001
High (%)	3717 (14.0)	2831 (30.3)		4366 (13.4)	2762 (30.6)	
Total physical activity (METs/week)	34.1 ± 6.7	33.7 ± 6.7	< 0.001	32.7 ± 5.7	33.2 ± 5.8	< 0.001
Sleep duration ≤ 5 h^†^ (%)	462 (1.7)	618 (6.6)	< 0.001	976 (3.0)	858 (9.5)	< 0.001
Skipping breakfast^†^ (%)	2705 (10.3)	2663 (28.8)	< 0.001	3457 (10.7)	2357 (26.6)	< 0.001
Energy intake (kcal/day)	2185 ± 670	2198 ± 713	0.114	1883 ± 624	1893 ± 666	0.206
Vegetables and fruits (g/day)	398.3 ± 231.6	357.9 ± 223.4	< 0.001	499.1 ± 245.2	481.8 ± 253.3	< 0.001
Vegetables (g/day)	211.4 ± 135.6	189.2 ± 126.5	< 0.001	245.1 ± 140.0	233.8 ± 141.7	< 0.001
Fruits (g/day)	187.5 ± 156.0	169.4 ± 153.4	< 0.001	254.9 ± 178.5	249.2 ± 191.8	0.010
Fish (g/day)	95.3 ± 56.6	93.1 ± 60.4	0.002	91.1 ± 51.5	89.1 ± 53.4	0.001
Meat (g/day)	65.1 ± 43.4	68.0 ± 45.6	< 0.001	58.3 ± 38.9	61.0 ± 42.7	< 0.001
Fibre (g/day)	12.6 ± 4.8	11.3 ± 4.6	< 0.001	14.3 ± 4.7	13.7 ± 4.9	< 0.001
Sodium (mg/day)	5049 ± 1607	4953 ± 1700	< 0.001	4943 ± 4658	4923 ± 1584	0.507

Values are expressed as mean ± standard deviation or number (%).

*Determined by t-test or Chi-square test.

^†^Participants with missing information were excluded (living arrangement n = 1485; smoking status: n = 4981; job type: n = 13,737; working hours: n = 6462; perceived mental stress: n = 529; Sleep duration: n = 345; skipping breakfast: n = 1425).

*BMI* body mass index, *MET* metabolic equivalent task.

We confirmed 3,763 stroke cases (2184 men and 1579 women) and 878 CHD cases (626 men and 252 women) during a mean follow-up duration of 13.3 years. The results of age- and study area-adjusted and multivariable-adjusted HRs (95% CIs) of incident CVD according to the self-reported irregular daily routine stratified by sex are shown in Table 2. Significant positive associations were found between an irregular daily routine and risk of total CVD and total stroke in the age- and study area-adjusted models in men. However, these significant associations disappeared after further adjustment for living arrangement, alcohol intake, smoking status, mental stress, working hours, job type, breakfast, sleep duration, BMI, METs, and dietary factors. In women, multivariable adjustment attenuated the significant associations between an irregular daily routine and increased risk of total CVD, total stroke, and CHD, but the following positive associations remained significant: total CVD (HR 1.24, 95% CI 1.11–1.39), total stroke (HR 1.21, 95% CI 1.07–1.36), and CHD (HR 1.49, 95% CI 1.11–2.00). These results were almost the same after adjustment for possible mediator variables, such as hypertension, hypercholesterolaemia, and diabetes mellitus (Table S1). When we performed a sensitivity analysis after excluding cases that occurred within 3 years after the baseline, significant associations were observed between an irregular daily routine and increased risks of total CVD (HR 1.25, 95% CI 1.10–1.41), total stroke (HR 1.19, 95% CI 1.04–1.36), and CHD (HR 1.60, 95% CI 1.17–2.20) in women.

**Table 2:** Age- and study area-adjusted and multivariable-adjusted hazard ratios and 95% confidence intervals of incident cardiovascular diseases according to self-reported irregular daily routine.

	Men		p values	Women		p values
	Regular	Irregular		Regular	Irregular	
	HR	HR (95% CI)		HR	HR (95% CI)	
Number of participants	26,801	9409		32,822	9083	
Person-years	346,384	120,252		448,668	122,793	
**Total cardiovascular disease**						
Number of cases	2095	715		1387	444	
Age- and study area-adjusted model	1.0 (Ref.)	1.15 (1.06–1.26)	0.001	1.0 (Ref.)	1.30 (1.16–1.44)	< 0.001
Multivariable-adjusted model*	1.0 (Ref.)	1.05 (0.96–1.15)	0.314	1.0 (Ref.)	1.24 (1.11–1.39)	< 0.001
**Total stroke**						
Number of cases	1628	556		1204	375	
Age- and study area-adjusted model	1.0 (Ref.)	1.16 (1.05–1.28)	0.004	1.0 (Ref.)	1.25 (1.11–1.41)	< 0.001
Multivariable-adjusted model*	1.0 (Ref.)	1.06 (0.95–1.17)	0.288	1.0 (Ref.)	1.21 (1.07–1.36)	0.003
**Coronary heart disease (myocardial infarction or sudden cardiac death)**						
Number of cases	467	159		183	69	
Age- and study area-adjusted model	1.0 (Ref.)	1.14 (0.95–1.37)	0.155	1.0 (Ref.)	1.59 (1.20–2.10)	0.001
Multivariable-adjusted model*	1.0 (Ref.)	1.02 (0.84–1.24)	0.840	1.0 (Ref.)	1.49 (1.11–2.00)	0.008

*Adjusted by age and study area, quartile of BMI, living arrangement, alcohol intake, cigarette smoking status, perceived mental stress, working hours, job type, frequency of eating breakfast, sleep duration, quartile of METs, energy intake, energy-adjusted dietary intakes of vegetables, fruits, meat, fish, and sodium.

*HR* hazard ratio, *CI* confidence interval, *BMI* body mass index, *MET* metabolic equivalent task.

To evaluate the interaction between an irregular daily routine and vegetable and fruit consumption on CVD risk, stratified analyses with cross-product terms were also conducted (Table 3). We observed a positive and significant association between an irregular daily routine and the risk of total CVD only in the population with vegetable and fruit consumption lower than the median value in men (HR 1.15, 95% CI 1.02–1.29) and detected an interaction between an irregular daily routine and vegetable and fruit consumption (p for interaction = 0.032). Similarly, multivariable HRs for total stroke and CHD in the lower vegetable and fruit consumption groups were slightly higher in men with irregular daily routine than in those with regular routine, but the interactions were not statistically significant in total stroke (p for interaction = 0.109) and CHD (p for interaction = 0.119). In women with lower vegetable and fruit consumption, multivariable HRs (95% CI) of total CVD and stroke were 1.32 (1.14–1.54) and 1.36 (1.15–1.60), respectively, and a significant interaction between an irregular daily routine and vegetable and fruit consumption was detected for total stroke (p for interaction = 0.039), but not for total CVD (p for interaction = 0.231). With regard to CHD in women, while multivariable HR (95% CI) in the higher vegetable and fruit consumption group was 1.90 (1.29–2.80), the interaction was not significant (p for interaction = 0.073).

**Table 3:** Hazard ratios for incident cardiovascular diseases according to self-reported irregular daily routine: stratified analysis according to vegetable and fruit consumption.

	Men		p for interaction	Women		p for interaction
	Regular	Irregular		Regular	Irregular	
	HR	HR (95% CI)		HR	HR (95% CI)	
**Total cardiovascular disease**						
Vegetable and fruit consumption						
Lower group						
Person-years	166,457	68,126		220,879	64,630	
Number of cases	939	412		673	239	
Multivariable-adjusted model*	1.0 (Ref.)	1.15 (1.02–1.29)		1.0 (Ref.)	1.32 (1.14–1.54)	
Higher group			0.032			0.231
Person-years	179,927	52,126		227,789	58,164	
Number of cases	1156	303		714	205	
Multivariable-adjusted model*	1.0 (Ref.)	0.95 (0.83–1.08)		1.0 (Ref.)	1.16 (0.99–1.36)	
**Total stroke**						
Vegetable and fruit consumption						
Lower group						
Number of cases	744	321		576	210	
Multivariable-adjusted model*	1.0 (Ref.)	1.14 (0.99–1.31)		1.0 (Ref.)	1.36 (1.15–1.60)	
Higher group			0.109			0.039
Number of cases	884	235		628	165	
Multivariable-adjusted model*	1.0 (Ref.)	0.97 (0.84–1.13)		1.0 (Ref.)	1.06 (0.89–1.26)	
**Coronary heart disease**						
Vegetable and fruit consumption						
Lower group						
Number of cases	195	91		97	29	
Multivariable-adjusted model*	1.0 (Ref.)	1.18 (0.91–1.52)		1.0 (Ref.)	1.14 (0.74–1.75)	
Higher group			0.119			0.073
Number of cases	272	68		86	40	
Multivariable-adjusted model*	1.0 (Ref.)	0.88 (0.67–1.16)		1.0 (Ref.)	1.90 (1.29–2.80)	

*Adjusted by age, study area, quartile of BMI, living arrangement, alcohol intake, cigarette smoking status, perceived mental stress, working hours, job type, frequency of eating breakfast, sleep duration, quartile of METs, energy intake, energy-adjusted dietary intakes of meat, fish, and sodium.

*HR* hazard ratio, *CI* confidence interval, *BMI* body mass index, *MET* metabolic equivalent task.

In the analyses stratified by other demographic factors, such as BMI, living arrangement, smoking and drinking habits, working hours, total physical activity, perceived mental stress, sleep duration, and skipping breakfast, no significant interaction with an irregular daily routine was detected (Table S2), while multivariable HRs (95% CI) were 1.16 (1.01–1.33) in men and 1.50 (1.17–1.93) in women with the job type category ‘office work, profession, or other work’.

## Discussion

This study aimed to elucidate the association between the self-reported irregular daily routine and the risk of incident CVD in a middle- and older-aged population-based prospective study. We found that an irregular daily routine is significantly associated with an increased risk of CVD in women but not in men. Moreover, in the stratified analysis on total vegetable and fruit consumption, significant interactions were found for total CVD in men and total stroke in women. Notably, higher vegetable and fruit consumption might weaken the positive association between irregular daily routine and CVD risk.

No previous study has investigated the associations between a self-reported irregular daily routine and CVD risk in middle- and older-aged men and women. Those who reported their daily routine as irregular had characteristics such as long working hours, higher perceived mental stress, short sleep, and skipping breakfast. Some findings, mainly related to sleep schedules, have suggested that circadian misalignments could increase CVD risk. For example, a previous cross-sectional study, using actigraphy-assessed rest-activity cycle, has demonstrated that more regular activity rhythms were associated with lower odds of metabolic syndrome, hypertension, dyslipidaemia, and CVD^31^. Recent studies have also indicated that irregular sleep duration and timing increased the risk of cardiometabolic factors or CVD events^14–16,32,33^. Increasing cardiometabolic risks were also observed among other specific populations or settings with circadian misalignments, such as rotating-shift workers^10,11,34^, daylight-saving time^35^, and individuals with higher social jetlag^36–38^. Moreover, greater eveningness, which is closely related to a larger degree of irregular daily routine^39–41^, has been associated with an increased risk of CVD mortality^42^. These findings on circadian misalignments support our results in examining the association between a self-reported irregular daily routine and CVD risk.

The association between a self-reported irregular daily routine and the risk of CVD may be explained by several potential physiological mechanisms underlying them. Given the characteristics of those who reported their daily routine as irregular, it is assumed that the 24-h behavioural cycles, such as the sleep–wake and feeding schedules, are not aligned with endogenous circadian rhythms^4^. Previous studies have indicated an association between exposure to experimentally-imposed circadian misalignments and increased blood pressure^4,6^. Other studies have shown that day-to-day circadian misalignments are related to metabolic risk factors, such as a lower high-density lipoprotein-cholesterol level, higher triglyceride level, fasting plasma insulin level, insulin resistance, and adiposity^7,14^. Considering that our results were almost the same after adjustment for possible mediator variables, such as hypertension, hypercholesterolaemia, and diabetes mellitus (although we should note that the adjustment could be inadequate because these mediators were self-reported variables), other potential candidates should also be discussed. For example, elevated inflammatory markers, such as serum interleukin-6, C-reactive protein, and tumour necrosis factor-α, have been observed among those with circadian misalignments^5,6^. Given that low-grade inflammation is not negligible^43,44^ and may also be involved in pathways between shift workers and CVD risk^45,46^, inflammation may become one of the targeted factors to address the positive association between an irregular daily routine and the risk of CVD. Moreover, since it is well recognised that the autonomic nervous system^47^ and thrombogenic properties, such as platelet aggregability^48^ and plasminogen activator inhibitor 1^49^, have a robust circadian rhythm, these related factors may remain possible mechanisms between an irregular daily routine and the risk of CVD. Although the extent of these effects may be less than the impact of circadian misalignments caused by day-night reversal, the cumulative effects of an irregular daily routine (i.e., slight circadian misalignments) on health status would be noteworthy.

Our results show the possibility that the positive association between irregular daily routine and total CVD or total stroke may be weakened among men or women with a higher amount of vegetable and fruit consumption, indicating significant multiplicative interactions. A previous study has demonstrated the association between higher vegetable and fruit consumption and lower levels of inflammatory markers^50^, which are closely related to stroke and CHD^51,52^. Moreover, more pro-inflammatory diets may partially explain the increased inflammation-related chronic disease risk among those with an irregular daily routine due to day-night reversal^53^. Therefore, assuming that our population with a higher vegetable and fruit consumption had lower circulating levels of inflammatory markers, those with higher vegetable and fruit consumption may weaken the positive associations between an irregular daily routine and CVD or stroke among men and women. Although further investigations are warranted to understand the interaction (or effect modification) between an irregular daily routine and other potential factors of CVD, these angles may be important for those who inevitably have an irregular daily routine due to socio-environmental reasons. On the other hand, there was a tendency of interaction between an irregular daily routine and vegetable and fruit consumption on the risk of CHD among women, although it was not significant. However, the number of CHD cases in women was relatively small, and the confidence interval was especially wide for women in the higher vegetable and fruit consumption group.

In the present study, the lack of a positive association between an irregular daily routine and CVD risk among men could be partially explained by the results of stratified analysis by job type. The percentage of men with ‘office work, profession, or other work’ (excluding farming, forestry, fishery, self-employed) was twice that of women, and men with ‘homemaker or unemployed’ was one-fifth that of women, indicating a difference in the distribution of job type. Therefore, when we performed stratified analysis by job type, significant positive associations between an irregular daily routine and CVD risk were consistent in both men and women with job type ‘office work, profession, or other work’. However, since the detailed structures of circadian misalignments strongly depend on working environments, further research reflecting the current socio-environmental conditions among men and women is necessary.

The percentage of participants who ate breakfast every day was significantly lower in men and women who considered themselves to have an irregular daily routine in this study. A previous study reported that habitual skipping breakfast was associated with increased levels of inflammation^54^. Additionally, the frequency of breakfast intake was inversely associated with the risk of haemorrhagic stroke^17^. These studies suggest the importance of taking into account circadian misalignments between biological rhythms, sleep–wake cycles, and temporal irregularity in dietary habits. In fact, individuals with a more irregular pattern of energy intake, especially at breakfast, have an increased cardiometabolic risk^55,56^. Therefore, further studies are needed to confirm the validity of a self-reported irregular daily routine by objective measurements and examine the effects of dietary and sleep irregularity separately.

This study has several limitations. First, some participants might have changed their daily routines during the follow-up period. However, the percentage of individuals with the same response to the question, ‘Is your daily routine or activity schedule regular?’ in both the 5-year and 10-year follow-up surveys was 80.6%, and the Spearman rank correlation coefficients between the two surveys were 0.45 and 0.43 in men and women, respectively (data not shown). Second, the validity of a self-reported irregular daily routine was not confirmed. A self-reported irregular daily routine report may contain some ambiguity. Since this may have involved the association between an irregular daily routine and the risk of CVD toward the null in men and away from null in women, estimates without a bias could be obtained by objectively assessing what a self-reported irregular daily routine reflects (e.g. circadian misalignment due to weekday-weekend differences, day-to-day variation over a week, and the timing of dietary intake). Moreover, as inter-individual variation in lifestyles, which was included in the multivariable-adjusted model, would reflect presence/absence of irregular daily routine to a certain degree, the multivariable model could explain how it influences CVD risk. Especially, the fact that multivariable adjustment did not attenuate the association in women indicates that unmeasured lifestyle or unknown factors related to irregularity, such as circadian misalignment, would result in an increased risk of CVD development in women. However, an irregular daily routine was associated with poorer sleep quality in the 10-year follow-up data (i.e. longer sleep latency, arousal during night sleep, and early morning awakening) (data not shown), which corresponds well to the deleterious effect of circadian misalignment^4,57^. Therefore, although the validity of a self-reported irregular daily routine may not be low, a well-validated exposure is desirable^58^. Third, the assessment of an irregular daily routine was based on binary data. Further research on the effects of different degrees of irregularity should be more meaningful. Fourth, the results stratified by demographic characteristics should be interpreted as exploratory because analyses with several stratified variables may give rise to the potential for type I error inflation due to multiplicity issues. Finally, although we measured and adjusted for several confounders, there could still be potential residual confounding due to unmeasured variables, such as rotating shift work.

## Conclusion

This prospective cohort study revealed that a self-reported irregular daily routine was significantly and positively associated with the risk of incident CVD, especially in women, and that these significant associations may be weakened in the populations with higher vegetable and fruit consumption. Daily routine as well as vegetable and fruit intake might be considered for the prevention of CVD in the middle-aged and older populations.

## Supplementary Information

Supplementary Tables.

## Data Availability

The datasets used and/or analysed during the current study are available from the corresponding author on reasonable request.
