# Supplementary material for: Association between irregular daily routine and risk of incident stroke and coronary heart disease in a large Japanese population
Source: Sci Rep. 2022 Sep 21;12:15750. doi: 10.1038/s41598-022-20019-8 (PMC9492773; doi:10.1038/s41598-022-20019-8)
Supplement: Supplementary file 1 — Supplementary Tables. [file 41598_2022_20019_MOESM1_ESM.docx]

**Supplemental Material (Online supplemental data for publication)**

Association between irregular daily routine and risk of incident stroke and coronary heart disease in a large Japanese population

Takahiro Yoshizaki,^1^ Junko Ishihara,^2*^ Ayaka Kotemori,^2,3^ Yoshihiro Kokubo,^4^ Isao Saito,^5^ Hiroshi Yatsuya,^6^ Kazumasa Yamagishi,^7^ Norie Sawada,^3^ Motoki Iwasaki,^3^ Hiroyasu Iso,^8^ Shoichiro Tsugane,^3^ and the JPHC Study Group^a^

**Table S1. Age- and study area-adjusted and multivariable-adjusted hazard ratios and 95% confidence intervals of incident cardiovascular diseases according to self-reported irregular daily routine**

|  | | Men | | | p values |  | Women | | | p values |
| --- | --- | --- | --- | --- | --- | --- | --- | --- | --- | --- |
|  | | Regular |  | Irregular |  |  | Regular |  | Irregular |  |
|  | | HR |  | HR (95% CI) |  |  | HR |  | HR (95% CI) |  |
| Total cardiovascular disease | |  |  |  |  |  |  |  |  |  |
|  | Multivariable-adjusted model^*^ | 1.0 (Ref.) |  | 1.03 (0.94–1.13) | 0.514 |  | 1.0 (Ref.) |  | 1.24 (1.10–1.39) | <0.001 |
| Total stroke | |  |  |  |  |  |  |  |  |  |
|  | Multivariable-adjusted model^*^ | 1.0 (Ref.) |  | 1.04 (0.94–1.15) | 0.472 |  | 1.0 (Ref.) |  | 1.20 (1.06–1.36) | 0.003 |
| Coronary heart disease | |  |  |  |  |  |  |  |  |  |
| (Myocardial infarction or sudden cardiac death) | | |  |  |  |  |  |  |  |  |
|  | Multivariable-adjusted model^*^ | 1.0 (Ref.) |  | 1.01 (0.83–1.22) | 0.942 |  | 1.0 (Ref.) |  | 1.46 (1.09–1.96) | 0.012 |

^*^Adjusted by age and study area, quartile of BMI, living arrangement, alcohol intake, cigarette smoking status, perceived mental stress, working hours, job type, frequency of eating breakfast, sleep duration, quartile of METs, energy intake, energy-adjusted dietary intakes of vegetables, fruits, meat, fish, sodium, hypertension, hypercholesterolaemia, and diabetes mellitus.

HR, hazard ratio; CI, confidence interval; BMI, body mass index; MET, metabolic equivalent task

**Table S2. Hazard ratios for incident cardiovascular disease according to self-reported irregular daily routine: Stratified analysis according to demographic characteristics**

|  | Men | | | p for interaction |  | Women | | | p for interaction |
| --- | --- | --- | --- | --- | --- | --- | --- | --- | --- |
|  | Regular |  | Irregular |  |  | Regular |  | Irregular |  |
|  | HR |  | HR (95% CI)^*^ |  |  | HR |  | HR (95% CI)^*^ |  |
| **BMI**^†^ |  |  |  |  |  |  |  |  |  |
| Lean | 1.0 (Ref.) |  | 1.45 (0.90–2.33) | 0.195 |  | 1.0 (Ref.) |  | 0.89 (0.44–1.82) | 0.320 |
| Normal | 1.0 (Ref.) |  | 1.05 (0.94–1.18) | - |  | 1.0 (Ref.) |  | 1.29 (1.11–1.50) | - |
| Obese | 1.0 (Ref.) |  | 1.02 (0.87–1.19) | 0.710 |  | 1.0 (Ref.) |  | 1.25 (1.05–1.48) | 0.755 |
| **Living alone** |  |  |  |  |  |  |  |  |  |
| Yes | 1.0 (Ref.) |  | 1.10 (0.74–1.64) | 0.775 |  | 1.0 (Ref.) |  | 1.17 (0.81–1.70) | 0.737 |
| No | 1.0 (Ref.) |  | 1.03 (0.94–1.14) |  |  | 1.0 (Ref.) |  | 1.25 (1.11–1.41) |  |
| **Smoking** |  |  |  |  |  |  |  |  |  |
| Never | 1.0 (Ref.) |  | 1.08 (0.91–1.28) | 0.865 |  | 1.0 (Ref.) |  | 1.32 (1.17–1.49) | 0.309 |
| Smoker | 1.0 (Ref.) |  | 1.06 (0.95–1.18) |  |  | 1.0 (Ref.) |  | 1.07 (0.73–1.58) |  |
| **Drinking** |  |  |  |  |  |  |  |  |  |
| Never | 1.0 (Ref.) |  | 1.17 (1.00–1.37) | 0.107 |  | 1.0 (Ref.) |  | 1.22 (1.08–1.37) | 0.237 |
| Drinker | 1.0 (Ref.) |  | 1.00 (0.90–1.12) |  |  | 1.0 (Ref.) |  | 1.45 (1.10–1.92) |  |
| **Working hours**^†^ |  |  |  |  |  |  |  |  |  |
| <5 h | 1.0 (Ref.) |  | 0.98 (0.79–1.22) | 0.398 |  | 1.0 (Ref.) |  | 1.15 (0.95–1.40) | 0.318 |
| ≥5 h and <9 h | 1.0 (Ref.) |  | 1.09 (0.96–1.23) | – |  | 1.0 (Ref.) |  | 1.31 (1.10–1.55) | - |
| ≥9 h | 1.0 (Ref.) |  | 1.02 (0.85–1.23) | 0.561 |  | 1.0 (Ref.) |  | 1.31 (0.97–1.76) | 0.998 |
| **Type of job**^†^ |  |  |  |  |  |  |  |  |  |
| Farming, forestry, fishery, self-employed | 1.0 (Ref.) |  | 0.91 (0.76–1.09) | 0.602 |  | 1.0 (Ref.) |  | 1.24 (0.88–1.75) | 0.473 |
| Office work, profession, other work | 1.0 (Ref.) |  | 1.16 (1.01–1.33) | 0.266 |  | 1.0 (Ref.) |  | 1.50 (1.17–1.93) | 0.030 |
| Homemaker, unemployed | 1.0 (Ref.) |  | 0.99 (0.77–1.27) | – |  | 1.0 (Ref.) |  | 1.08 (0.92–1.27) | – |
| **Total physical activity** |  |  |  |  |  |  |  |  |  |
| Lower than the median value | 1.0 (Ref.) |  | 1.16 (1.01–1.32) | 0.057 |  | 1.0 (Ref.) |  | 1.30 (1.10–1.54) | 0.683 |
| Higher than the median value | 1.0 (Ref.) |  | 0.96 (0.83–1.11) |  |  | 1.0 (Ref.) |  | 1.24 (1.03–1.49) |  |
| **Perceived mental stress** |  |  |  |  |  |  |  |  |  |
| Low or middle | 1.0 (Ref.) |  | 1.04 (0.94–1.16) | 0.775 |  | 1.0 (Ref.) |  | 1.25 (1.10–1.42) | 0.709 |
| High | 1.0 (Ref.) |  | 1.08 (0.88–1.31) |  |  | 1.0 (Ref.) |  | 1.19 (0.93–1.52) |  |
| **Short sleeper** |  |  |  |  |  |  |  |  |  |
| Yes | 1.0 (Ref.) |  | 1.25 (0.83–1.88) | 0.373 |  | 1.0 (Ref.) |  | 1.24 (0.81–1.89) | 0.974 |
| No | 1.0 (Ref.) |  | 1.04 (0.94–1.14) |  |  | 1.0 (Ref.) |  | 1.25 (1.11–1.40) |  |
| **Skipping breakfast** |  |  |  |  |  |  |  |  |  |
| Yes | 1.0 (Ref.) |  | 0.99 (0.81–1.21) | 0.532 |  | 1.0 (Ref.) |  | 1.49 (1.13–1.96) | 0.162 |
| No | 1.0 (Ref.) |  | 1.06 (0.96–1.17) |  |  | 1.0 (Ref.) |  | 1.20 (1.06–1.36) |  |

^*^Adjusted by age, study area, quartile of BMI, living arrangement, alcohol intake, cigarette smoking status, perceived mental stress, working hours, job type, frequency of eating breakfast, sleep duration, quartile of metabolic equivalent task (METs), energy intake, energy–adjusted dietary intakes of meat, fish, and sodium (except for stratification variables).

^†^Reference group for interaction was normal BMI, working ≥5 h and <9 h, or homemaker and unemployed.

HR, hazard ratio; CI, confidence interval; BMI, body mass index
